# Estimation of Ground Contact Time with Inertial Sensors from the Upper Arm and the Upper Back

**DOI:** 10.3390/s23052523

**Published:** 2023-02-24

**Authors:** Leticia González, Antonio M. López, Diego Álvarez, Juan C. Álvarez

**Affiliations:** Electrical Engineering Department, Campus of Gijon, University of Oviedo, 33204 Oviedo, Spain

**Keywords:** running, ground contact time, inertial measurement unit (IMU)

## Abstract

Ground contact time (GCT) is one of the most relevant factors when assessing running performance in sports practice. In recent years, inertial measurement units (IMUs) have been widely used to automatically evaluate GCT, since they can be used in field conditions and are friendly and easy to wear devices. In this paper we describe the results of a systematic search, using the Web of Science, to assess what reliable options are available to GCT estimation using inertial sensors. Our analysis reveals that estimation of GCT from the upper body (upper back and upper arm) has rarely been addressed. Proper estimation of GCT from these locations could permit an extension of the analysis of running performance to the public, where users, especially vocational runners, usually wear pockets that are ideal to hold sensing devices fitted with inertial sensors (or even using their own cell phones for that purpose). Therefore, in the second part of the paper, an experimental study is described. Six subjects, both amateur and semi-elite runners, were recruited for the experiments, and ran on a treadmill at different paces to estimate GCT from inertial sensors placed at the foot (for validation purposes), the upper arm, and upper back. Initial and final foot contact events were identified in these signals to estimate the GCT per step, and compared to times estimated from an optical MOCAP (Optitrack), used as the ground truth. We found an average error in GCT estimation of 0.01 s in absolute value using the foot and the upper back IMU, and of 0.05 s using the upper arm IMU. Limits of agreement (LoA, 1.96 times the standard deviation) were [−0.01 s, 0.04 s], [−0.04 s, 0.02 s], and [0.0 s, 0.1 s] using the sensors on the foot, the upper back, and the upper arm, respectively.

## 1. Introduction

Ground contact time (GCT) is defined as the amount of time a runner is in contact with the ground for each step (from initial foot contact to final foot contact). GCT has been addressed as one of the most influential biomechanical factors that affect running economy [1,2].

The use of inertial measurement units (IMUs) for the measurement and evaluation of sports or human motion, has been an established technique for a long time [3]. The possibility of using these sensors outside of the laboratory environment has made them interesting for application in localization [4], occupational health and safety [5], or pathological gait analysis [6], among others. This has led, in the past decade, to an exploration of the possibility of estimating GCT from IMUs attached to different parts of an athlete’s body.

**Figure 1:**
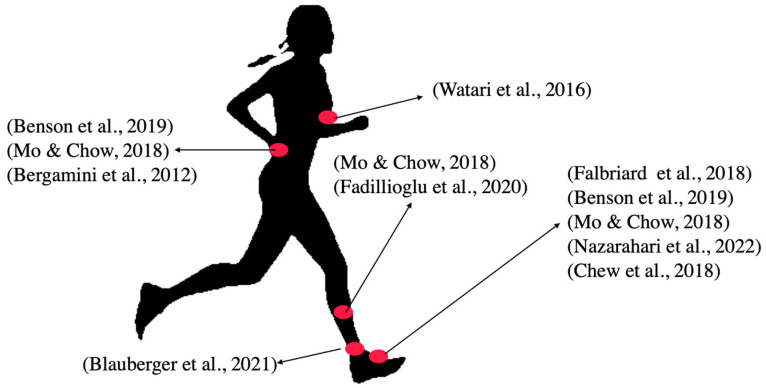
IMU location of the reference works, selected from the state of the art, as a result of a systematic search. References: (Falbriard et al., 2018), [7]; (Benson et al., 2019), [8]; (Mo & Chow, 2018), [9]; (Nazarahari et al., 2022), [10]; (Chew et al., 2018), [11]; (Blauberger et al., 2021), [12]; (Fadillioglu et al., 2020), [13]; (Bergamini et al., 2012), [14]; (Watari et al., 2016), [15].

Different body segments have been selected for the placement of the sensors, and different algorithms have been proposed to estimate the GCT from the sampled signals. From a systematic search of the literature, described below, we have found (see Figure 1) relevant works based on sensors placed on the lower body segments: the foot [7,8,9,10,11], the ankle [12], the tibia [9,13], the pelvis [8,9], and the trunk lower back [14]. However, the upper body has received little attention in the scientific literature as a target place to locate the sensors for the estimation of GCT, with a few exceptions, where GCT was estimated from a torso-mounted IMU [15].

The upper body is, from a practical point of view, an ideal place for wearable sensor placement. In fact, runners, especially vocational runners, mainly motivated by the necessity of wearing their cellular phones during sports practice, usually wear pockets attached to the upper back or to the upper arm that are ideal to hold sensing devices. Therefore, a correct estimation of GCT from such places could permit an extension of the analysis of the performance of their running to the public, even allowing them to do this from their own cellular phone, that is in general provided with inertial sensors, and could be easily provided with an app to record the sampled signals and even to estimate GCT. Therefore, in this work we conduct an experimental study to estimate the GCT from an inertial sensor located at the upper back and the upper arm. We include advanced (semi-elite) and amateur runners (six in total), running on a treadmill at different rhythms. We compare the performance between our estimations and those obtained from estimations based on a ground truth optical motion capture system (Optitrack). For assessment purposes, we also address the estimation of the GCT from an IMU placed at the foot, using a validated method proposed in the state of the art [7].

The paper is organized as follows. In Section 2 we describe the design, execution, and results of a systematic search based on the Web of Science, to select and analyze the most relevant works for GCT estimation from body-worn IMUs. Section 3 and Section 4 describe the experimental methods and results. The paper is finished with the discussion and conclusions in Section 5.

## 2. State of the Art

A systematic search was run using the Web of Science database on the 4th of February 2022. The following sentence was used for that purpose:


        TS = ( ( ( (foot OR initial OR terminal OR ground) NEAR/3 (contact) )
        OR
        (“toe off”)
        OR
        (gait NEAR/3 event*)
         )
        AND
        (acceleromet* or inertial or gyroscop* or IMU)
        AND (run*)
         )
        AND
       (DT==(“ARTICLE”)))
	   

The inclusion criteria for the prior screening of the references were:

- Conference papers were discarded;
- The primary objective of the study had to be the timing of step events or ground contact time as a summarized result. Works with a different primary objective were not considered, even though event detection was addressed for that purpose. This consideration was applied sequentially over the title, abstract, and the whole paper, to screen the recorded papers.

After this preliminary screening, 11 papers were selected for a more exhaustive screening. From these, two papers were later discarded, as they only addressed identification of the initial contact event [16,17].

**Figure 2:**
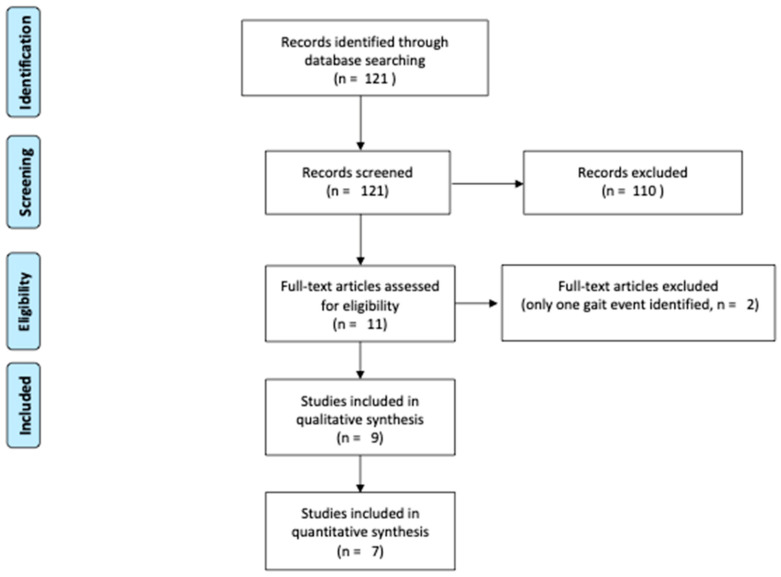
Flow diagram of the study according to the PRISMA methodology.

Figure 2 summarizes the process using the PRISMA (Preferred Reporting Items for Systematic Reviews and Meta-analyses) flow diagram.

After the references screening, two researchers independently analyzed each reference and findings were discussed later. The reading was focused on:

- The identification of the location of the IMU on the body;
- The identification of the participants in the experiments (number of people, running experience, gender, velocities, and duration);
- The identification of the performance of the method described to estimate the GCT with respect to the gold standard method used for validation of the results (accuracy, precision, or similar performance metrics).

Two papers were removed in this phase [10,13]. While they addressed the identification of initial and final contact events, they did not make a paired analysis that could permit the calculation of the GCT.

Table 1 contains the information extracted from the selected references. For outdoor experiments, optical [14], photoelectric bar [12], and force platforms [9] systems were used as the gold standard. Indoor experiments (four from seven) used an optical MOCAP system [11] or an instrumented treadmill as the gold standard.

Elite participants were only considered in two studies from the seven. In the rest, recreational runners were involved in the experiments, mixing male and female runners. We include in Table 1 the number of runners for each paper that were used for the GCT estimation, and whose results were validated against the gold standard. This refers to the 25 subjects defined as the validation set in [7], and the 14 subjects that were validated against the gold standard in [8]. With regards to [14], experiments were performed with amateur and elite runners. We include results from the elite athletes that were used to test the estimation algorithms. For the rest of the analyzed references, all the subjects involved in the experiments were considered.

Recreational runners run at recreational running velocities, except for [7], where higher running rhythms, up to 5.56 m/s (20 km/h, 3 min/km), were considered.

GCT estimation performance is reported in the different works, using different metrics. For our study, we decided to interpret the information reported in the different papers using a generic measure of accuracy and precision. To interpret accuracy and precision values (those included in Table 1), we adopted the following decisions:

- Positive values for the accuracy were used to indicate that GCT estimations from the IMU are higher than GCT estimations from the ground truth (negative values were used in the other case);
- In [7], the central tendency and dispersion of estimation errors are indicated, respectively, using (i) the median of the mean error, and (ii) the median of the standard deviation, between the IMU and the force-platform-based GCT estimations in the different trials (person/speed). We have taken these values as being representative of the accuracy and precision of estimations;
- Accuracy and precision were compiled from [8,12] and [14] using Bland–Altman plots between the accelerometer-based and the gold-standard-based identification of GCT. We used the offset as accuracy. Variability is reported in these works in terms of 95% LoA (we have interpreted this as 1.96 times the standard deviation unless a different value was specified in the paper). Values included in our table refer to the standard deviations, and are calculated from them. In [14], the included values were identified from a figure, so perhaps a little error may exist in the values included in the table;
- The authors of [9] directly reported the mean and standard deviation of the error from each IMU-based method and the ground truth;
- The authors of [15] reported the average accuracy and LoA for errors from estimations compared to a force platform at the different velocities. We include in the table the median of these values, as representatives of the accuracy and precision of the method. Similarly, [11] reports average accuracy and precision values for errors from estimations compared to a force platform at the different velocities. We include in the table the median of these values as representative values.

## 3. Materials and Experimental Methods

### 3.1. Experiments

**Figure 3:**
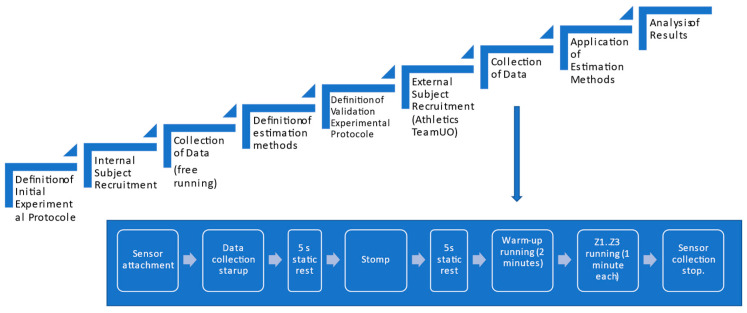
Experimental flowchart.

Experiments were conducted on a treadmill. Two rounds of experiments were developed (see Figure 3 for a graphical description of the experimental procedures).

A preliminary experimental round, involving three volunteer adults, recruited from the scientific team (one female, two male, age 41 ± 14.52 years, weight 75.33 ± 8.39 kg, height 175 ± 8.66 cm), were used to define the estimation methods. Subjects ran at a comfortable running pace, and cameras and IMU signals were collected and matched to define the estimation methods from the IMUs.

The validation experiment involved six healthy adults from the University of Oviedo athletics team (two female, four male, age 37 ± 12.38 years, weight 60.17 ± 8.84 kg, height 168.67 ± 10.19 cm) without any symptomatic musculoskeletal injuries. Two of them were semi-elite runners. Written informed consent was obtained in advance from all the participants. Runners were told to warm-up at their desired running pace for two minutes. After that, they ran at three different rhythms (Z1, Z2, Z3), one minute for each. Velocities corresponding to each rhythm were specified by their coach attending to the personal conditions of each one (see Table 2).

**Figure 4:**
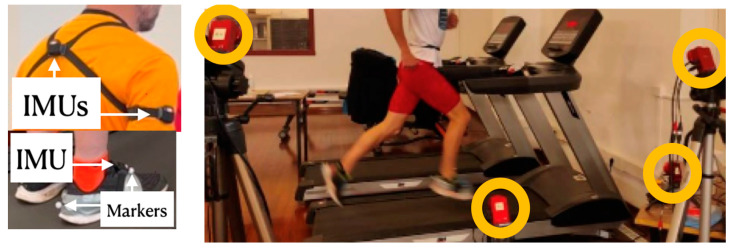
(**Left**): positioning of the IMUs, and the reflective markers for the optical MOCAP system, over the body of the athlete. (**Right**): Optitrack camera disposition.

Runners were equipped with Xsens DOT V2 inertial sensors (see Figure 4, left). Two of them were firmly attached, using elastic bands, to the upper back and the upper arm of the subjects. An additional sensor was attached to the right foot, close to the third metatarsal, using an adhesive band. The sampling frequency was fixed at 100 Hz. Sensor measurements between the different IMUs were synchronized using the option available in the configuration software provided by the manufacturer, Xsens DOT app for Android. Sensors were also configured to store the sampled data in their internal memory, to avoid data loss. Data were transferred to a personal computer once the experiments were completed.

Three Flex 3 Optitrack (Figure 4, right) cameras were positioned so that the markers on the athlete’s right foot were always visible. Reflective markers were placed on the heel and the third metatarsal of the foot (Figure 4, left-bottom), positioned so as not to impede the correct running technique. The four cameras were connected via a USB to a personal computer, on which the Optitrack Motive Tracker 2 software was run. An additional Flex 3 camera was used to record the image of the foot for testing purposes.

Synchronization between the IMU system and the Optitrack was performed manually for each runner. The Optitrack system and the IMUs were each started on their own time base. After initiating the data collection in both systems, the athlete remained static for 5 s and then tapped their foot vertically on the treadmill, remaining static for another 5 s. The time of contact of the foot with the floor, visually identified from the corresponding signal peaks in both sensor signals, was used as the initial time, with all other events measured with respect to it.

### 3.2. GCT Estimation from the Optical System

The position signals measured by the MOCAP system were filtered by a bidirectional FIR filter, designed using the window method, with order 6 and a cutoff frequency of 3 Hz.

The steps were segmented from the vertical marker position signal located on the toe of the foot. Local minima, with a prominence of at least 40 mm, were located on this marker and used as step markers. To detect initial and final contacts, a threshold was placed at each step, corresponding to 20 mm above the recorded minimum of the toe of the foot (we observed that the minimum of the marker at the heel systematically occurred later). The instant at which this value was reached was identified as IC, while the instant at which this height was exceeded again was identified as FC.

### 3.3. GCT Estimation from the Inertial Sensors

**Figure 5:**
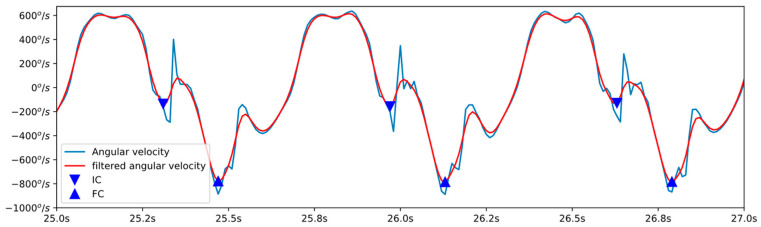
Blue line: angular velocity in the mid-lateral axis obtained from the foot-mounted inertial sensor. Red line: low pass filtered angular velocity. IC and FC events are indicated by a downward triangle and an upward triangle, respectively.

Based on previous work [7], the initial and final foot contacts can be detected from the angular velocity of the pitch of a foot-mounted inertial sensor. As reported, three local minima can be identified in each footfall cycle (see Figure 5), the first of which corresponds to the start of contact and the second to the end of contact.

To detect these minima, the angular velocity signal was filtered using the same bidirectional FIR filter used for the camera signals. All local minima and maxima present in this filtered signal were detected. Those where the slew rate was positive were discarded. Maxima were used to segment the signal into single steps.

In each cycle (step) of the filtered signal, three minima appear. The first of the minima present is identified as IC, while the absolute minimum of the cycle is identified as FC.

**Figure 6:**
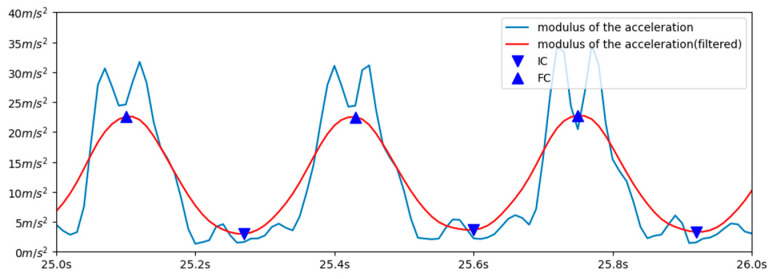
Blue line: modulus of the acceleration obtained from the upper-back-mounted inertial sensor. Red line: low pass filtered acceleration. IC and FC events are indicated by a downward triangle and an upward triangle, respectively.

In our training experiments, we found that the initial and final foot contact events can be identified from the minimum and maximum, respectively, of the low pass filtered resulting acceleration, recorded by the inertial sensor mounted on the upper back (see Figure 6). For this purpose, the modulus of the acceleration at each instant of time was calculated. This signal was then filtered with a bidirectional filter, of moving average and order 20.

Since the signals vary in amplitude in different cycles, a dynamic threshold was established, which was used to differentiate the absolute maximum of each cycle from the rest of the maxima present. The threshold was taken as the average between the maximum and the minimum value recorded in the last 50 measurements.

Each time a local maximum was encountered, if the acceleration value was higher than the set threshold, the maximum was identified as the final contact of the step.

The initial contact was identified as the local minimum detected just before the FC (see Figure 6).

**Figure 7:**
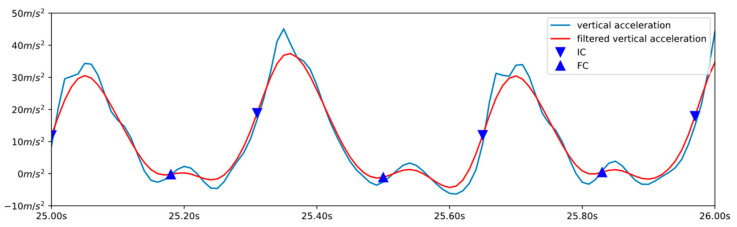
Blue line: vertical acceleration obtained from the upper-arm-mounted inertial sensor. Red line: low pass filtered vertical acceleration. IC and FC events are indicated by a downward triangle and an upward triangle, respectively.

Finally, we proposed a method to estimate the GCT from the IMU mounted on the upper arm, estimating the initial contact as the maximum of the jerk in the vertical axis (which corresponds to the inflexion point of the vertical acceleration), and the final contact from the local minima of the derivative of the resulting acceleration (see Figure 7).

For this purpose, the accelerations were filtered using a bidirectional moving average filter of order 10.

The step cycle was segmented using the resulting acceleration signal. For this purpose, a dynamic threshold was set as the mean between the maximum and minimum of the last 50 samples, and the steps were segmented considering the maximum of the resulting acceleration exceeding this value.

The initial contact of each step was located as the last maximum of the vertical jerk acceleration signal before the maximum of the resulting acceleration.

The final contact of each step was located as the first minimum of the vertical acceleration after the maximum of the resulting acceleration.

In all cases, we sought to achieve a logical sequence of detections, in which each IC was followed by its corresponding FC. If any of the algorithms detected two ICs in a row, or two FCs in a row, the second one was eliminated, to maintain the logical sequence of events.

On the other hand, while the MOCAP system and the sensor located on the foot only detected the events corresponding to the right foot, the inertial sensors located above the waist (on the back and on the arm) detected the initial and final contacts of both feet. To make a comparison between the different methods, the events corresponding to the left foot were not considered for further analysis.

## 4. Experimental Results

A total of 1722 steps were identified from the cameras, 1708 from the foot-attached IMU, 3421 from the back-attached IMU, and 3414 from the arm-attached IMU. From the arm and back, a much higher number of steps were identified, since the steps corresponding to both the right and left foot were detected, while from the camera and from the foot, only those of the right foot were detected.

To perform step-by-step paired comparisons, the times recorded as the initial contact from the steps identified from the cameras, and from each of the IMUs, were checked. Each of the steps identified from the camera was matched to the step recorded by each of the IMUs closest in time. Steps from the IMUs not matched to steps from the cameras were discarded. The total number of steps matched was 1705 from the foot IMU, and 1720 from each of the arm and back IMUs. Finally, we discarded those steps where either method provided clearly erroneous results, including step times of less than 15 ms or contact times of less than 8 ms. A total of 1673 steps were finally included in the statistical study. In summary, more than 99% of the steps were identified from all the IMUs (99.8% from the back and arm sensors). Likewise, 98.1% of the total number of detected steps presented consistent step times and contact times, and were included in the statistical study.

Table 3 shows (mean ± std) the GCT estimated for every subject from the different sensors, and the corresponding step time estimated from the ground truth. An ANOVA analysis revealed that the mean GCT estimated from the cameras was significatively different for each subject and running velocity (p~0). The test also revealed that the mean estimation errors from the camera, and from each of the IMUs, were significantly different (p~0) for the different IMUs, subjects, and running velocities, with the exception of estimation errors from the upper arm, where a *p*-value of 0.066 was found for the influence of the running velocity.

**Figure 8:**
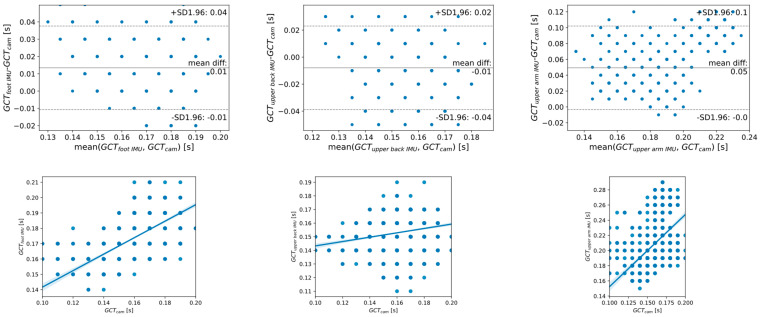
(**Top**). Bland–Altman plot for the error in the estimation of GCT from the cameras and from the IMUs: foot-attached (**left**), upper-back-attached (**center**) and upper-arm-attached (**right**). The paired difference between the GCT estimations from the IMUs and the cameras are plotted against their mean for all the steps in the experiments. Mean difference in the GCT estimation between the IMU and the cameras is plotted with a central continuous straight line. 95% limits of agreement (1.96 times the standard deviation of the estimation errors) are plotted with upper and lower dashed straight lines. (**Bottom**). Correlation plots. *X*-axis shows the GCT estimated from the cameras. *Y*-axis shows the corresponding GCT estimated from the IMUs attached to the foot (**left**), upper back (**center**), and upper arm (**right**).

Figure 8 shows the Bland–Altman plot for the error analysis in the estimations from the cameras compared to each of the accelerometers. The average difference for estimations from the cameras, and both from the foot and the upper back IMUs, is 0.01 s (one sample) as an absolute value (a positive bias means that the IMU overestimates the cameras and a negative difference means that the IMU underestimates the cameras). This average error grows to 0.05 s (five samples) for the difference between the estimation from the cameras and from the upper-arm-attached IMU. The LoA (1.96 times the standard deviation) was [−0.01 s, 0.04 s] (−1 to 4 samples) from the foot-attached sensor, [−0.04 s, 0.02 s] (−4 to 2 samples) from the upper back sensor, and [0.0 s, 0.1 s] (0 to 10 samples) from the upper arm IMU. Figure 8 shows also correlation plots that relate the GCT estimated from the cameras and the different IMUs. A positive relation is found in all the cases.

## 5. Discussion

A great deal of work has been done in recent years addressing the estimation of GCT from inertial sensors placed on different body segments. To have a clear picture of the state of the art, in the initial phase of our study we addressed a systematic search from one of the widest and most recognized databases of scientific research (Web of Science), finding related works. We found that, usually lower body segments have been selected for sensor placement. The upper body has rarely been considered for that purpose, with the exceptions of some sporadic studies that considered a sensor placed on the trunk. This motivated our experimental work, motivated by the potential benefits of estimating GCT from these positions, that may extend the improvement of running technique to the public, permitting them to do this even from consumer devices such as cellular phones.

To design the experiments, we adopted the usual configuration for indoor studies based on treadmill running. In our case, an instrumented treadmill was not available, so we used a standard treadmill and a gold standard visual MOCAP system, as proposed in previous work [11]. Methods to estimate GCT from the upper back and the upper arm sensors were designed in advance, from an initial study considering unstructured experiments involving three amateur subjects. For the validation experiments, we included a mixture of recreational (four) and semi-elite (two) runners, with the aim of extending our results to a wide population. Running velocities in our experiments (3.3 m/s to 5.6 m/s) were in the range of normal to high rhythms (2.78 m/s to 5.56 m/s), as proposed in [7].

The IMUs were placed in comfortable positions on the upper back and arm, inspired by the usual commercially available utility pockets (mainly used for mobile phones), in the form of armbands around the upper arm, or harnesses on the upper back. A third IMU was placed on the foot, as a representative of the state of the art, IMU-based GCT estimation methods. This third IMU was used to interpret the results of the novel methods proposed in our work in the light of the results of other methods tested in the state of the art. With this paired analysis, we tried to mitigate the effect of the different experimental conditions from our setup to those used in the original experiments, including the actual sensors used. The work in [7] was selected as representative for several reasons. In the first place, the original work considered the largest population and the widest range of velocities, from the references analyzed. Most importantly, the algorithm was based on simple techniques to detect the initial and final contacts. Described events in the signal were clearly found in our signals, without complex processing, and therefore estimation from the reproduced algorithm was expected to be less prone to error implementation than could be the case from other algorithms analyzed.

GCT estimation requires a prior step segmentation from the sampled signals. The success of this phase was not generally reported in the analyzed references from the systematic search, with the exception of [8], that reported a step rate detection of 97%, and [12], that reported values close to 100% for step detection success. Our results also show a high step detection rate from all the IMUs, identifying above 98% of the actual steps identified by the optical system.

Using the estimations from the optical system, we found (Table 3) an average step time of 0.32 s and an average GCT from the cameras of 0.18 s. These results are in agreement with previous studies for treadmill running [18], where, for velocities between 12 km/h and 20 km/h (our velocity range), the authors reported an average step time of 0.34 s and an average GCT of 0.22 s.

The GCT estimated from the IMU attached to the foot showed an average bias of 0.014 s and a precision of 0.013 s. These values signify that, on average, we get an estimation error of one sample with a precision of one additional sample. This is an acceptable error, as an improvement over this would suppose nearly perfect detection. This result confirms that the estimation from the accelerometers works properly in our experimental setup. The results in our experiments differ from the results reported in the original work that used the same estimation method applied to a foot-worn inertial sensor [7], which reported a higher estimation error accuracy, of −0.03 s, with a precision of 0.004 s, and [11] reported an improved estimation accuracy of −0.008 s, with a precision of ±0.004 s. The experimental conditions are different in our setup, which may have caused the differences.

We found an average error value of 0.008 s in the estimations from the upper-back-attached sensor compared to the ground truth, with a precision (standard deviation) of 0.016 s. This error is similar to that found from estimation from the foot-attached sensor, that may lead one to suspect that a similar performance may be expected in general terms between the foot-attached and the upper-back-attached IMUs, although the difference was found to be significant in statistical terms (ANOVA, *p*~0). Regarding estimations from the upper arm, GCT estimations were less accurate and precise, reporting up to 0.049 s of average error with a precision of 0.027 s (standard deviation). However, the values are in the range of reported values for GCT estimation from the state of the art. The authors of [7] and [9] reported average errors around 0.03 s, and [8] and [9] reported a similar average error, around 0.05 ms. A precision with an estimated standard deviation around 30 ms was less frequent. Only [9] reported a similar standard deviation of error, of 34.1 ms, for estimation errors from a shank-mounted inertial sensor.

**Figure 9:**
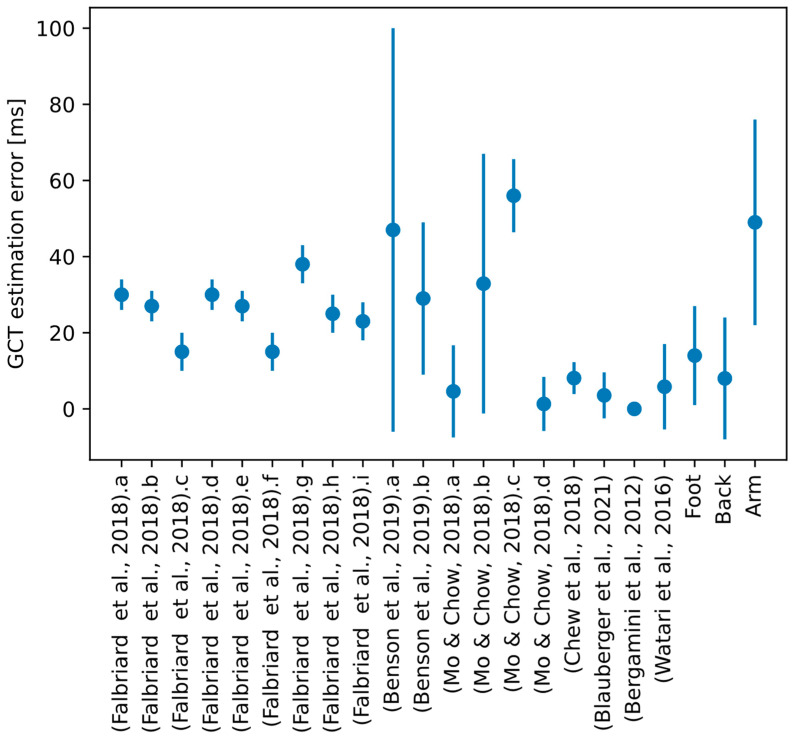
Error bars (accuracy ± precision) reported in the references analyzed from the state of the art and the methods proposed in this paper. X-axis labels show the reference and a letter (as indicated in Table 1) referencing the specific estimation method when different techniques are proposed in the same work. The last three values correspond to the methods proposed in this paper (using IMUs at the foot, the upper back, and the upper arm). References: (Falbriard et al., 2018), [7]; (Benson et al., 2019), [8]; (Mo & Chow, 2018), [9]; (Chew et al., 2018), [11];(Blauberger et al., 2021), [12]; (Bergamini et al., 2012), [14]; (Watari et al., 2016), [15].

As a summary, Figure 9 shows, using error bars, the precision (as absolute values, to facilitate the comparison) and accuracy of the methods reported in the state of the art and those analyzed in this paper. Varied performance values have been reported. There are, on the one side, very accurate and precise methods [12,14], and estimations from the pelvis and the foot, performed by [9]. This high performance can be justified, as in these studies experimental conditions were very controlled, which may have reduced the variability of the steps analyzed. In [12], 50 m of stable running were monitored from elite athletes; the authors of [9] and [14] analyzed only a reduced number of steps (ten and four, respectively). In any case, the estimation method seems to affect the results, as [9] reports, from the same experiments, an improved performance for estimations based on the pelvis and a combination of the shank and the foot. For the remaining references, performance is varied, and the methods analyzed in this paper (shown in orange on the right) are comparable to them, with estimations from the foot and the upper back having among the lowest errors and, on the contrary, estimations from the upper arm among the highest.

This work is, to our knowledge, the first experimental study to estimate GCT from the upper arm and the upper back. The results confirm the feasibility of obtaining a significant estimation of GCT from the upper back and the upper arm. In line with previous studies [7], further studies addressing a systematic assessment of other upper back/arm signal events, as indicators of the initial and final foot contact, may eventually confirm the optimality of the proposed algorithm, or lead to improvements of the estimation results. Tests with a greater population, and outside laboratory conditions, are other lines of future research.

## Figures and Tables

**Table 1:** Systematic search results. From left to right: reference, gold standard method used for validation of estimated GCT, number of participants (M: men, W: women), category of runners (recreational, elite, intermediate), speeds considered in the experiments, location of the sensor (different methods were addressed in some works from a single sensor), accuracy and precision of estimated GCT.

Reference	Gold Standard	Participants	Cat	Speed	Location	GCT Estimation Accuracy	GCT Estimation Precision
[7]	Instrumented treadmill (force platform)	25 M/W	Intermediate	2.78 m/s to 5.56 m/s, 0.56 m/s variation, 30 s each	Foot (method a)	−30 ms	4 ms
					Foot (method b)	−27 ms	4 ms
					Foot (method c)	−15 ms	5 ms
					Foot (method d)	−30 ms	4 ms
					Foot (method e)	−27 ms	4 ms
					Foot (method f)	−15 ms	5 ms
					Foot (method g)	−38 ms	5 ms
					Foot (method h)	−35 ms	5 ms
					Foot (method i)	−23 ms	5 ms
[8]	Instrumented treadmill (force platform)	8 M, 4 W	Recreational	2.7, 3.2, and 3.6 m/s 90 s each	Foot (method a)	47 ms	53 ms
					Pelvis (method b)	−29 ms	20 ms
[12]	Photoelectric bars (outdoor)	5	Elite	100 m sprint, 50 m were monitored	Ankle	3.55 ms	6.04 ms
[9]	Force platform (outdoor)	7 M, 4 W	--	4.1 ± 1.2 m/s 10 steps were analyzed from each runner	Pelvis (method a)	4.6 ms	12.1 ms
					Shank (method b)	32.9 ms	34.1 ms
					Foot (method c)	−56.0 ms	9.6 ms
					Shank+foot (method d)	−1.3 ms	7.1 ms
[15]	Instrumented treadmill (force platform)	14 M, 8 W	Intermediate	2.7, 3.0, 3.3, 3.6, 3.9 m/s 30 s each	Torso	−5.82 ms	11.21 ms
[11]	Optical MOCAP	10 M	--	2.22, 2.5, 2.78, 3.06 m/s 3 min each	Foot	−8.09 ms	4.19 ms
[14]	High speed camera (outdoor)	5	Elite	6 sprint (4 steps per sprint)	Trunk	0.002 ms	0.01 ms

**Table 2:** Race paces in km/h (m/s) for each of the athletes, as specified by their coach.

Runner	Z1	Z2	Z3
1	17 (4.7)	18.5 (5.1)	20 (5.6)
2	14 (3.9)	15 (4.2)	16 (4.4)
3	13 (3.6)	14 (3.9)	15 (4.2)
4	12.5 (3.5)	13 (3.6)	14.5 (4.0)
5	12 (3.3)	13.5 (3.7)	15 (4.2)
6	16.5 (4.6)	18 (5)	19.5 (5.4)

**Table 3:** Ground contact time in seconds (mean ± std) for the different subjects using the different sensors. The right-hand column shows aggregated results for the step time identified using the cameras.

	Camera	IMU Foot	IMU Upper Back	IMU Upper Arm	Step Time
Subject 1	0.145 ± 0.010	0.159 ± 0.010	0.151 ± 0.009	0.176 ± 0.009	0.316 ± 0.011
Subject 2	0.157 ± 0.017	0.167 ± 0.008	0.146 ± 0.007	0.212 ± 0.020	0.314 ± 0.011
Subject 3	0.165 ± 0.011	0.182 ± 0.008	0.160 ± 0.006	0.205 ± 0.007	0.317 ± 0.012
Subject 4	0.166 ± 0.014	0.172 ± 0.007	0.145 ± 0.013	0.196 ± 0.010	0.316 ± 0.014
Subject 5	0.172 ± 0.007	0.190 ± 0.008	0.158 ± 0.007	0.258 ± 0.025	0.321 ± 0.009
Subject 6	0.159 ± 0.012	0.174 ± 0.010	0.157 ± 0.006	0.204 ± 0.013	0.331 ± 0.004

## References

[1] di Michele R., Merni F. (2014). The concurrent effects of strike pattern and ground-contact time on running economy. J. Sci. Med. Sport.

[2] Moore I.S., Ashford K.J., Cross C., Hope J., Jones H.S.R., McCarthy-Ryan M. (2019). Humans Optimize Ground Contact Time and Leg Stiffness to Minimize the Metabolic Cost of Running. Front. Sport. Act. Living.

[3] Gouwanda D., Senanayake S.M.N.A. Emerging Trends of Body-Mounted Sensors in Sports and Human Gait Analysis. Proceedings of the 4th Kuala Lumpur International Conference on Biomedical Engineering 2008.

[4] González R.C., López A.M., Rodriguez-Uría J., Álvarez D., Alvarez J.C. (2010). Real-time gait event detection for normal subjects from lower trunk accelerations. Gait Posture.

[5] Álvarez D., Alvarez J.C., González R.C., López A.M. (2016). Upper limb joint angle measurement in occupational health. Comput. Methods Biomech. Biomed. Engin..

[6] Glowinski S., Blazejewski A., Krzyzynski T. (2017). Inertial Sensors and Wavelets Analysis as a Tool for Pathological Gait Identification. Innovations in Biomedical Engineering. Advances in Intelligent Systems and Computing.

[7] Falbriard M., Meyer F., Mariani B., Millet G.P., Aminian K. (2018). Accurate Estimation of Running Temporal Parameters Using Foot-Worn Inertial Sensors. Front. Physiol..

[8] Benson L.C., Clermont C.A., Watari R., Exley T., Ferber R. (2019). Automated accelerometer-based gait event detection during multiple running conditions. Sensors.

[9] Mo S., Chow D.H.K. (2018). Accuracy of three methods in gait event detection during overground running. Gait Posture.

[10] Nazarahari M., Khandan A., Khan A., Rouhani H. (2022). Foot angular kinematics measured with inertial measurement units: A reliable criterion for real-time gait event detection. J. Biomech..

[11] Chew D.K., Ngoh K.J.H., Gouwanda D., Gopalai A.A. (2018). Estimating running spatial and temporal parameters using an inertial sensor. Sport. Eng..

[12] Blauberger P., Horsch A., Lames M. (2021). Detection of ground contact times with inertial sensors in elite 100-m sprints under competitive field conditions. Sensors.

[13] Fadillioglu C., Stetter B.J., Ringhof S., Krafft F.C., Sell S., Stein T. (2020). Automated gait event detection for a variety of locomotion tasks using a novel gyroscope-based algorithm. Gait Posture.

[14] Bergamini E., Picerno P., Pillet H., Natta F., Thoreux P., Camomilla V. (2012). Estimation of temporal parameters during sprint running using a trunk-mounted inertial measurement unit. J. Biomech..

[15] Watari R., Hettinga B., Osis S., Ferber R. (2016). Validation of a torso-mounted accelerometer for measures of vertical oscillation and ground contact time during treadmill running. J. Appl. Biomech..

[16] Aubol K.G., Milner C.E. (2020). Foot contact identification using a single triaxial accelerometer during running. J. Biomech..

[17] Reenalda J., Zandbergen M.A., Harbers J.H.D., Paquette M.R., Milner C.E. (2021). Detection of foot contact in treadmill running with inertial and optical measurement systems. J. Biomech..

[18] Ogueta-Alday A., Morante J.C., Rodríguez-Marroyo J.A., García-López J. (2013). Validation of a New Method to Measure Contact and Flight Times During Treadmill Running. J. Strength Cond. Res..

